# Hyperpolarization-Activated Cyclic Nucleotide-Gated Non-selective (HCN) Ion Channels Regulate Human and Murine Urinary Bladder Contractility

**DOI:** 10.3389/fphys.2018.00753

**Published:** 2018-06-19

**Authors:** Felix Mader, Steffen Müller, Ludwig Krause, Armin Springer, Karoline Kernig, Chris Protzel, Katrin Porath, Simone Rackow, Tristan Wittstock, Marcus Frank, Oliver W. Hakenberg, Rüdiger Köhling, Timo Kirschstein

**Affiliations:** ^1^Oscar Langendorff Institute of Physiology, University of Rostock, Rostock, Germany; ^2^Department of Medical Biology, Electron Microscopy Center, University of Rostock, Rostock, Germany; ^3^Department of Urology, University of Rostock, Rostock, Germany

**Keywords:** ZD7288, 18β-glycyrrhetic acid, HCN1 knockout, electron microscopy, organ bath

## Abstract

**Purpose:** Hyperpolarization-activated cyclic nucleotide gated non-selective (HCN) channels have been demonstrated in the urinary bladder in various species. Since they play a major role in governing rhythmic activity in pacemaker cells like in the sinoatrial node, we explored the role of these channels in human and murine detrusor smooth muscle.

**Methods:** In an organ bath, human and murine detrusor smooth muscle specimens were challenged with the HCN channel blocker ZD7288. In human tissue derived from macroscopically tumor-free cancer resections, the urothelium was removed. In addition, HCN1-deficient mice were used to identify the contribution of this particular isoform. Expression of HCN channels in the urinary bladder was analyzed using histological and ultrastructural analyses as well as quantitative reverse transcriptase polymerase chain reaction (RT-PCR).

**Results:** We found that the HCN channel blocker ZD7288 (50 μM) both induced tonic contractions and increased phasic contraction amplitudes in human and murine detrusor specimens. While these responses were not sensitive to tetrodotoxin, they were significantly reduced by the gap junction inhibitor 18β-glycyrrhetic acid suggesting that HCN channels are located within the gap junction-interconnected smooth muscle cell network rather than on efferent nerve fibers. Immunohistochemistry suggested HCN channel expression on smooth muscle tissue, and immunoelectron microscopy confirmed the scattered presence of HCN2 on smooth muscle cell membranes. HCN channels seem to be down-regulated with aging, which is paralleled by an increasing effect of ZD7288 in aging detrusor tissue. Importantly, the anticonvulsant and HCN channel activator lamotrigine relaxed the detrusor which could be reversed by ZD7288.

**Conclusion:** These findings demonstrate that HCN channels are functionally present and localized on smooth muscle cells of the urinary bladder. Given the age-dependent decline of these channels in humans, activation of HCN channels by compounds such as lamotrigine opens up the opportunity to combat detrusor hyperactivity in the elderly by drugs already approved for epilepsy.

## Introduction

In an aging population, degenerative age-associated diseases will be present in an increasing proportion of patients in the future. In contrast to this, pharmacological innovations are tested in highly selected, healthy and young populations. The overactive bladder is a common disease with higher prevalence in the elderly (Madersbacher et al., 1998). On the morphological level, connective tissue was found to be increased at the cost of smooth muscle tissue (Lepor et al., 1992). On the functional level, however, the pathomechanisms involved in enhanced contractile activity are still poorly understood although a number of recent findings on non-adrenergic, non-cholinergic transmission (Toozs-Hobson et al., 2014) and pacemaker systems (Kubota et al., 2011) have accelerated the current notion of detrusor smooth muscle contraction mechanisms.

Since the discovery of pacemaker cells in the lamina propria of the urinary bladder that were similar to of interstitial cells of Cajal (ICC) found in the gastrointestinal tract (Wiseman et al., 2003), research on the role of these cells in bladder motility has attracted substantial attention. Now it is commonly accepted that the human bladder contains c-kit positive pacemaker cells involved in bladder motility (Andersson and McCloskey, 2014) that may be relevant targets for the treatment of overactive bladder (Juszczak et al., 2014).

The prototypical molecular basis of pacemaker cells is the hyperpolarization-activated cyclic nucleotide gated non-selective cation (HCN) channel. HCN channels are primarily expressed in nerve cells (Moosmang et al., 1999; Santoro et al., 2000; Lörincz et al., 2002; Notomi and Shigemoto, 2004), and sinoatrial node cells (Shi et al., 1999; Moosmang et al., 2001) where these channels have therefore attracted most scientific attention so far. In contrast, there are only very limited reports on HCN channel expression in the urogenital tract. One early study suggested the presence of an inwardly rectifying ZD7288-sensitive ion channel on rat detrusor smooth muscle cells (Green et al., 1996). The currently available data indicate that all four isoforms, HCN1-4, are expressed on ICC within the detrusor muscle and the mucosal layer of the urinary bladder (He et al., 2012; Xue et al., 2012; Kashyap et al., 2015). However, these studies also revealed species differences. While in human tissue HCN4 appeared to be the predominant subtype, rat tissue showed highest expression for HCN1 (He et al., 2012; Xue et al., 2012; Kashyap et al., 2015).

In the present study, we explored the role of HCN channels in urinary bladder contractility of mice and humans.

## Materials and Methods

### Human and Murine Detrusor Smooth Muscle Strips

Human detrusor smooth muscle strips were prepared from surgically resected human urinary bladders obtained from 40 patients with an average age of 70 ± 12 years (mean ± SD, range 36–86 years old; 27 male patients, 13 female patients) who underwent radical cystectomy for bladder cancer. While chemotherapy was occasionally initiated before surgery, radiotherapy was performed after the resection. Immediately following resection of the urinary bladder, a tissue sample of approximately 2 cm width was excised from the macroscopically unaffected wall of the bladder. All *in vitro* experiments with human material performed in this study were approved by the local ethics committee (University of Rostock), and informed consent was obtained from each patient.

Human detrusor samples were submerged in a storage solution containing (in mM) 120 NaCl, 4.5 KCl, 26 NaHCO_3_, 1.2 NaH_2_PO_4_, 1.6 CaCl_2_, 1.0 MgSO_4_, 0.025 Na_2_-EDTA, 5.5 glucose, 5 HEPES (pH = 7.4) for transport from the Department of Urology to the Institute of Physiology. They were freed from adipose tissue, urothelium and lamina propria and cut into 4–8 muscle strips of 1 cm length and 2–3 mm width.

Murine detrusor muscle strips were prepared from 46 wildtype C57BL6 mice and 32 HCN1^-/-^ mice (male and female mice; 290 ± 91 days old, mean ± SD; Charles River, Sulzfeld, Germany). To this end, the mice were anesthetized with diethyl ether and decapitated. The urinary bladder was quickly removed and immersed in storage solution as for human samples. One murine urinary bladder was inserted in toto in the organ bath.

In order to record isometric forces of human and murine detrusor muscle strips, thin nylon threads were sutured to either end of these strips to fix them longitudinally in an organ bath (Panlab ML0146/C, ADInstruments, Spechbach, Germany) filled with a buffer that contained (in mM) 120 NaCl, 4.7 KCl, 2.5 CaCl_2_, 1.2 MgCl_2_, 30 NaHCO_3_, 1.2 KH_2_PO_4_, 0.5 Na_2_-EDTA, 5.5 glucose, 2 sodium pyruvate (pH = 7.4) and was gassed with carbogen (95% O_2_ and 5% CO_2_).

### Isometric Contractions *in Vitro*

After fixation in the organ bath, the temperature was slowly raised to 37°C and the detrusor specimens were slightly stretched (up to 10 mN) and allowed to recover until a stable baseline tone with rhythmic activity was observed (typically 1 h in murine strips and up to 5 h in human strips). Isometric contractions or relaxations of the smooth muscle strips were measured by force transducers (MLT0201), recorded with a bridge amplifier (ML224) connected to an analog-to-digital converter (Powerlab 4/30, LabChart 7, ADInstruments).

**FIGURE 1:**
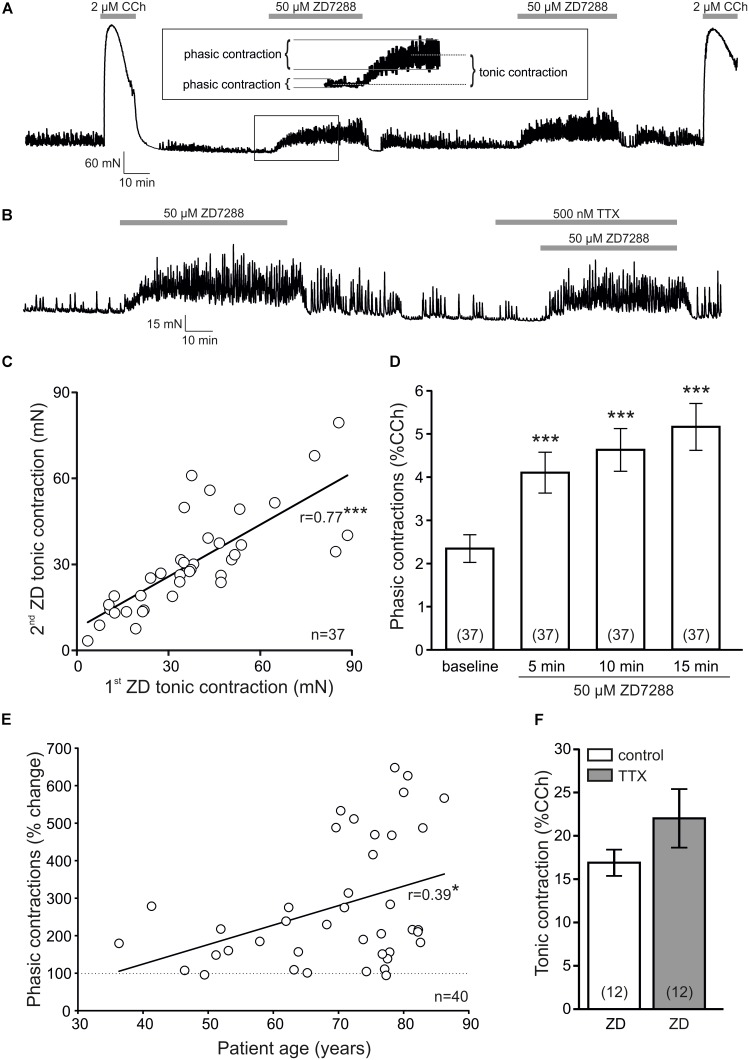
ZD7288 increases contractility of human bladder specimens. **(A)** Sample trace of an isometric force recording showing strong reaction upon 2 μM carbachol (CCh) and reproducible enhancement of both tonic and phasic contractions following 50 μM ZD7288. At the end of the experiment, the specimen was challenged again with CCh in order to demonstrate stable recording conditions. Phasic and tonic contractions are explained in the inset (enlarged from the sample trace, size 200%, indicated by the box). **(B)** Sample force recording showing that pre-treatment with 500 nM tetrodotoxin (TTX) did not alter ZD7288-induced contractility changes. **(C)** The 1st and 2nd ZD7288 application caused highly reproducible tonic contractions in human bladder specimens (*r* = 0.77, *P* < 0.001, paired *t*-test). **(D)** The increase of phasic contraction amplitudes after ZD7288 (in % CCh response) developed over a period of 15 min (*P* < 0.001, Wilcoxon signed rank test). **(E)** The increase of phasic contraction amplitudes after ZD7288 were positively correlated to patient age (*r* = 0.39, *P* < 0.05, paired *t*-test). **(F)** Tonic contractions following ZD7288 (in % CCh responses) were not altered by TTX pre-treatment.

The major aim of this study was to analyze the effect of the HCN channel blocker ZD7288 on bladder motility. ZD7288 was used at a concentration of 50 μM since pilot experiments revealed that 50 μM was the least concentration leading to reproducible results (EC50 value of ∼40 μM). The muscarinic agonist carbachol (CCh) was used as viability test at the beginning and the end of each experiment for normalization. Experiments were only included for statistical evaluation when the CCh response at the end of the experiment was at least 50% of the initial contraction. The baseline tone before drug application was defined as the mean isometric force over 2 min, and a tonic contraction or relaxation was defined as the change of this mean isometric force (dotted lines in **Figure 1A** inset). In the case of CCh, we recorded the maximal tonic contraction. In contrast, ZD7288-induced effects developed over 15 min, and therefore were analyzed at 5, 10, and 15 min. After 15 min, the ZD7288-induced effects were occasionally reduced and not further analyzed. The phasic contractions were defined as peak-to-peak contraction amplitude on a running average basis (gray lines in **Figure 1A** inset). To this end, we calculated the mean of all maxima and the mean of all minima within a period of 2 min. The phasic contraction amplitude was calculated as the difference between these mean values. All tonic or phasic contractions were expressed as the percentage of the initial CCh response to control for differences due to the various amounts of smooth muscle cells in the specimens. All n-numbers refer to the number of patients or the number of mice, respectively. Thus, all specimens from one subject were averaged and entered the subsequent statistical analysis as a single data point. Carbachol (CCh), ZD7288 (ZD), 18β-glycyrrhetic acid (18β-GA) and TTX were purchased from Tocris Bioscience (Bristol, United Kingdom). All other chemicals were obtained from Sigma-Aldrich (Taufkirchen, Germany). The application of drugs was performed by adding 100 μl to the organ bath solution (25 ml) to achieve the individual final concentration.

### Quantitative RT-PCR Analysis

Small pieces from the human detrusor smooth muscle (i.e., without urothelium or lamina propria) of approximately 1–2 mm width were prepared from the human tissue sample and immediately frozen in liquid nitrogen. Care was taken that these pieces for quantitative PCR were urothelium-free. For mRNA isolation, TRIZOL reagent was used, and total RNA was reverse-transcribed using Moloney murine leukemia virus reverse transcriptase (final concentration [Cf] = 10 U/μL) and RNasin Plus RNase inhibitor (Cf = 2 U/μL, both Promega Corporation, Madison, WI, United States) in the presence of random hexamers (Cf = 0.01 μg/μL) and dNTP Mix (Cf = 0.5 nmol/μL each, Invitrogen, Carlsbad, CA, United States). For the real-time PCR of the target genes (HCN1, HCN2, HCN3, and HCN4) as well as two standard reference genes (glyceraldehyde-3-phosphate dehydrogenase [GAPDH], β-actin [ACTB]), we used the QuantiFast SYBR Green PCR Kit (concentration as recommended by the manufacturer, Qiagen Inc., Valencia, CA, United States). The mastermix was aliquoted, cDNA (Cf = 2,5 ng/μl) and primers (Cf = 0.5 pmol/μL) were added. All target gene primers purchased from Molbiol (Berlin, Germany) are listed in **Table 1**, the PCR product length was 94-139 bp. The reference gene GAPDH was analyzed using the primers CCACTCCTCCACCTTTGAC (forward primer) and ACCCTGTTGCTGTAGCCA (reverse primer). ACTB was detected using Qiagen Primer Assays (Hs_ATCB_2_SG, QT01680476; Qiagen Inc., Valencia, CA, United States). Real-time PCR was performed using the ep mastercycler (software realplex 2.2, Eppendorf, Hamburg, Germany) with cycling parameters of 95.0°C for 2 min once, followed by 95.0°C for 15 s and the annealing temperature for 15 s, with normalized fluorescence read at 68.0°C (520 nm) for 40 cycles. The annealing temperatures were calculated using gradient PCR and set to 58.6°C for all PCRs. Single product amplification was confirmed by melting curve and gel electrophoresis analysis. Messenger-RNA (mRNA) expression levels were determined by normalizing the target genes (HCN1-4) with two standard reference genes (GAPDH, ACTB), expressed as 2^-ΔΔCt^ ± SEM.

**Table 1:** Forward and reverse primers of target genes HCN1-4.

Gene name	Forward primer	Reverse primer	PCR Product
HCN1	AGCAGCAGGTACAGC AGTCC	CGGGTCAGGTTGGTG TTGTG	139 bp
HCN2	GAATTCCATCCTCCTG CACAAG	CCATCTCGCGGTCG TACTTG	107 bp
HCN3	AGCACAGGAGCTCAG CTTAG	GCTGATGAGTCAGGGC AATG	112 bp
HCN4	GCTGATGAGTCAGGG CAATG	CGGTCATGCTGCACA ATCTG	94 bp


### Immunohistochemistry

Human detrusor samples (*n* = 7 patients, **Table 2**) were fixed in 4% phosphate-buffered paraformaldehyde for at least 1 day, cryoprotected in 30% sucrose for 3–5 days, and frozen in 2-methylbutane for long-term storage (-80°C). Horizontal sections (12 μm) were cut on a cryostat and treated with ice-cold acetone for 5–10 min, followed by incubation with the primary antibody (rabbit anti-HCN1 1:70, Alomone APC.056; mouse anti-HCN2 1:500, Abcam ab84817; rabbit anti-HCN4 1:2500, Abcam ab69054) at room temperature overnight. After several washes [4 × 5 min with phosphate-buffered saline (PBS)], sections were transferred to the secondary antibody solution (biotinylated goat anti-rabbit or anti-mouse IgG; Abcam kit) for 10 min at room temperature, followed by additional washes (4 × 5 min with PBS) and avidin-biotin-peroxidase complex solution for 10 min at room temperature. Antibody binding was detected after 10 min incubation with 3,3′ diaminobenzidine at room temperature (rabbit-specific or mouse-specific ABC Detection Kit, Abcam kit ab64261 and ab64259).

**Table 2:** Immunohistochemical expression of HCN isoforms in human specimens.

Patient #	Age (yrs)	HCN1	HCN2	HCN4
1	64	+ +	++	+
2	78	+ ++	+++	+ ++
3	85	+ +	+	+
4	54	+ +	0	0
5	74	+ +	+	+
6	75	+	0	0
7	68	+	+	+


### Transmission Electron Microscopy and Immunogold Labeling

Tissues were fixed in a solution of 4% paraformaldehyde in 0.1 M PBS (pH 7.3) for transmission electron microscopy (TEM) followed by washes in 0.1 M sodium phosphate buffer pH 7.3. Then specimens were dehydrated through an ascending series of ethanol prior to embedding in resin (LR white mittel, Plano, Wetzlar, Germany). Resin infiltration began with a 1:1 mixture of ethanol and resin overnight, followed by pure resin for 30 min and after medium change again for 4 h. After transfer to gelatine molds, the specimens were cured in an oven at 50°C for at least 24–48 h. Resin blocks were trimmed using a razor blade. Semithin sections (approximately 0.5 μm) and thin sections (approximately 70–90 nm) were cut with a Leica UC7r ultramicrotome using a diamond knife (Diatome Nidau, Switzerland). Semithin sections were mounted on glass slides and stained with an aqueous solution of 0.4% toluidine blue to visualize tissue morphology. Thin sections were mounted on formvar film-coated nickel grids and were contrasted with 7% aqueous uranyl acetate after immunogold labeling (see below). Ultrastructure was examined with a Zeiss EM902 electron microscope operated at 80 kV (Carl Zeiss, Oberkochen, Germany). Digital images were acquired with a side-mounted 1x2k FT-CCD Camera (Proscan, Scheuring, Germany) using iTEM camera control and imaging software (Olympus Soft Imaging Solutions, Münster, Germany).

For immunogold labeling studies all buffer solutions were sterile-filtered (0.2 μm). Grids were incubated on 40 μl droplets of the respective solutions placed on parafilm sheets. First, sections were moistened in electron microscopy phosphate-buffered saline (EM-PBS: 0.01 M sodium-phosphate buffer pH 7.4 containing 0.98% sodium chloride) and next were incubated in EM blocking buffer [EM-PBS containing 0.1% tween 20, 0.25% fish gelatin, 1% bovine serum albumin fraction V (EM buffer) supplemented with 2.5% normal goat serum and ovalbumin grade V] for 1 h at room temperature. Labeling of HCN2 was performed using a primary antibody (mouse anti-HCN2 1:100, Abcam ab84817; diluted in EM blocking buffer) at 4°C overnight. After several washes (5 × 10 min with EM-buffer), sections were incubated with a secondary antibody [goat anti-mouse IgG (H + L) coupled to 15 nm gold; BBI Solutions, Cardiff, United Kingdom; diluted in EM-buffer] 2 h at room temperature, followed by additional washes (2 × 10 min with EM-buffer, 3 × 10 min with EM-PBS) and rinses with distilled water.

### Statistics

All data are expressed as means ± SEM. For statistical comparisons, data were analyzed for normal distribution and then evaluated using adequate statistical tests for paired data sets (paired *t*-test or Wilcoxon signed rank test, respectively) and for unpaired data sets (unpaired *t*-test or Mann–Whitney test for comparisons of two groups, ANOVA with *post hoc* tests for comparisons of more than two groups) indicated in the text (SigmaStat 3.5). In the figures, the level of significance is indicated by asterisks ( ^+^*P* < 0.1; ^∗^*P* < 0.05; ^∗∗^*P* < 0.01; ^∗∗∗^*P* < 0.001).

## Results

### HCN Channel Inhibition Increases Human Detrusor Smooth Muscle Motility

The major goal of the present study was to explore the role of hyperpolarization-activated cyclic nucleotide-gated non-selective (HCN) ion channels in human urinary bladder contractile activity. To this end, we first tested the effect of the HCN channel blocker ZD7288 (50 μM) on spontaneous smooth muscle activity using human detrusor muscle strips freed from urothelium and lamina propria and that were fixed in an organ bath. After equilibration of up to 5 h in the organ bath, we regularly obtained a stable baseline tension and spontaneous phasic contractions. A typical sample trace of the first series of experiments is depicted in **Figure 1A**. At the beginning of the experiment, an initial strong contraction was induced by 2 μM carbachol (CCh, 10–15 min) in order to demonstrate viability of the muscle strip yielding 183 ± 13 mN (*n* = 40 patients with 150 specimens). After CCh washout and recovery of phasic activity after 45–60 min, the HCN channel blocker ZD7288 was applied and maintained for 30–40 min. ZD7288 caused both a tonic contraction and an increase of the amplitudes of phasic contractions (**Figure 1A**). In 37 cases, ZD7288 was repeated to test for reproducibility, and at the end of the experiment, the specimen integrity and maintenance was tested by challenging with CCh. We observed a high reproducibility of the tonic contraction resulting in a strong and significant correlation (*r* = 0.77, *P* < 0.001, *t*-test; **Figure 1C**; *n* = 37 patients with 138 specimens). Since isometric force responses may vary with differing amounts of smooth muscle cells, we normalized all responses to the initial CCh contraction to control for this variability. Thus, the amplitudes of phasic contractions increased over 15 min, and thus were analyzed after 5, 10, and 15 min (*P* < 0.001, Wilcoxon signed rank test; **Figure 1D**). The increase of phasic contraction amplitudes after 15 min in the presence of ZD7288 (5.2 ± 0.5%, *n* = 37) was significantly correlated to the equivalent increase following the second exposure to ZD7288 (5.6 ± 0.4%, *n* = 37; *r* = 0.54, *P* < 0.001, *t*-test) demonstrating a high reproducibility.

We next looked whether this tonic contraction or increase of phasic contraction amplitudes was dependent on patient age. However, there was no significant correlation between tonic contractions and age (*r* = 0.24, *P* = 0.133, *t*-test, *n* = 40 patients). In contrast, the amplitude enhancement of phasic contractions was positively correlated to patient age (*r* = 0.39, *P* < 0.05, *t*-test, *n* = 40 patients; **Figure 1E**).

Since HCN channels expressed on intrinsically active nerve fibers could be have contributed to the ZD7288 effects seen, we incubated the muscle strips with 500 nM tetrodotoxin (TTX) prior to exposure to ZD7288 in a subset of experiments (**Figure 1B**). The ZD-induced tonic contraction, however, was not significantly altered (22.0 ± 3.4% versus 16.9 ± 1.5%, *P* = 0.199, paired *t*-test, *n* = 12 patients with 24 specimens, **Figure 1F**). In addition, we did not observe a significant change in phasic contraction amplitudes in these experiments (baseline: 4.7 ± 1.1%, 15 min ZD7288: 5.2 ± 0.8%, *P* = 0.223, Wilcoxon signed rank test; *n* = 12 patients with 24 specimens). Hence, the tonic contraction and augmentation of phasic contractions by HCN channel inhibition was not mediated by intrinsically active nerve fibers, but probably due to a direct action on human detrusor smooth muscle cells. From **Figure 1B** it is also evident that ZD7288 could substantially increase the frequency of spontaneous phasic contractions. This effect was observed in 22/27 patients (with 61 specimens). On average, we found a significant increase from 2.8 ± 0.2 cycles/min to 3.5 ± 0.2 cycles/min (*P* < 0.001, paired *t*-test). Since an increase of the frequency of phasic contractions by ZD7288 has previously been reported in the rat detrusor (He et al., 2012; Deng et al., 2015), we focused on the ZD7288-mediated effects on contraction amplitudes.

**FIGURE 2:**
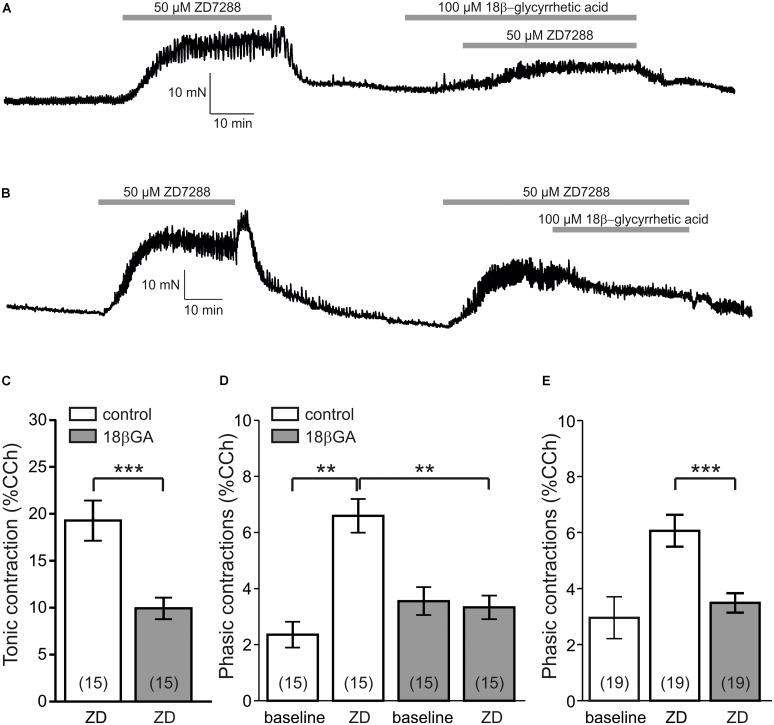
The gap junction blocker 18β-glycyrrhetic acid inhibits ZD7288-enhanced contractility. **(A)** Sample force recording showing that pre-treatment with 100 μM 18β-glycyrrhetic acid (18β-GA) inhibited alter ZD7288-induced contractility changes. **(B)** Pre-established ZD7288-enhanced contractility was sensitive to 18β-GA application. **(C)** Tonic contractions induced by ZD7288 were significantly reduced by 18β-GA pre-treatment (*P* < 0.001, paired *t*-test). **(D)** Phasic contraction amplitudes were significantly enhanced by ZD7288 (*P* < 0.01, Wilcoxon signed rank test). However, in the presence of 18β-GA, the ZD7288-induced increase was prevented. Note that the ZD7288-induced increase was significantly lower in 18β-GA incubation (*P* < 0.01, Mann–Whitney *U*-test). **(E)** Pre-established enhancement of phasic contractions following ZD7288 were significantly reduced by 18β-GA (*P* < 0.001, paired *t*-test).

Since smooth muscle cells are interconnected by gap junctions enabling transfer of membrane potential as well as calcium ions, we then tested the effect of the gap junction inhibitor 18β-glycyrrhetic acid (18β-GA, 100 μM) on ZD7288-induced changes of contraction amplitudes. In the first set of experiments, 18β-GA was applied prior to ZD7288 (**Figure 2A**). In these experiments, 18β-GA significantly reduced tonic contractions by ZD7288 (9.9 ± 1.1% vs. 19.3 ± 2.1%, *P* < 0.001, paired *t*-test; *n* = 15 patients with 38 specimens; **Figure 2C**). Moreover, the increase of phasic contraction amplitudes was abolished in the presence of 18β-GA (before: 3.6 ± 0.5%, 15 min ZD7288: 3.3 ± 0.4%; *P* < 0.01 versus control conditions, Mann–Whitney test; *n* = 15 patients with 38 specimens; gray bars in **Figure 2D**). With respect to the frequency of these phasic contractions, however, 18β-GA failed to block the ZD7288-induced increase (18β-GA: 4.0 ± 1.2 cycles/min; 18β-GA + ZD7288: 5.0 ± 1.1 cycles/min, *P* < 0.001, paired *t*-test).

Next, we performed another set of experiments adding 18β-GA to the established response to ZD7288 (**Figure 2B**). Here, we found a significant reduction of phasic contraction amplitudes (3.5 ± 0.3% vs. 6.1 ± 0.6%, *P* < 0.001, paired *t*-test, *n* = 19 patients with 47 specimens; **Figure 2E**) which suggests that gap junction activity contributed to the ZD7288-induced motility changes of human detrusor. Interestingly, under these conditions, 18β-GA further increased the frequency of phasic contractions (ZD7288: 4.2 ± 0.8 cycles/min; ZD7288 + 18β-GA: 4.7 ± 0.8 cycles/min, *P* < 0.005, paired *t*-test).

### HCN Channels Are Expressed on Human Detrusor Smooth Muscle Cells

**FIGURE 3:**
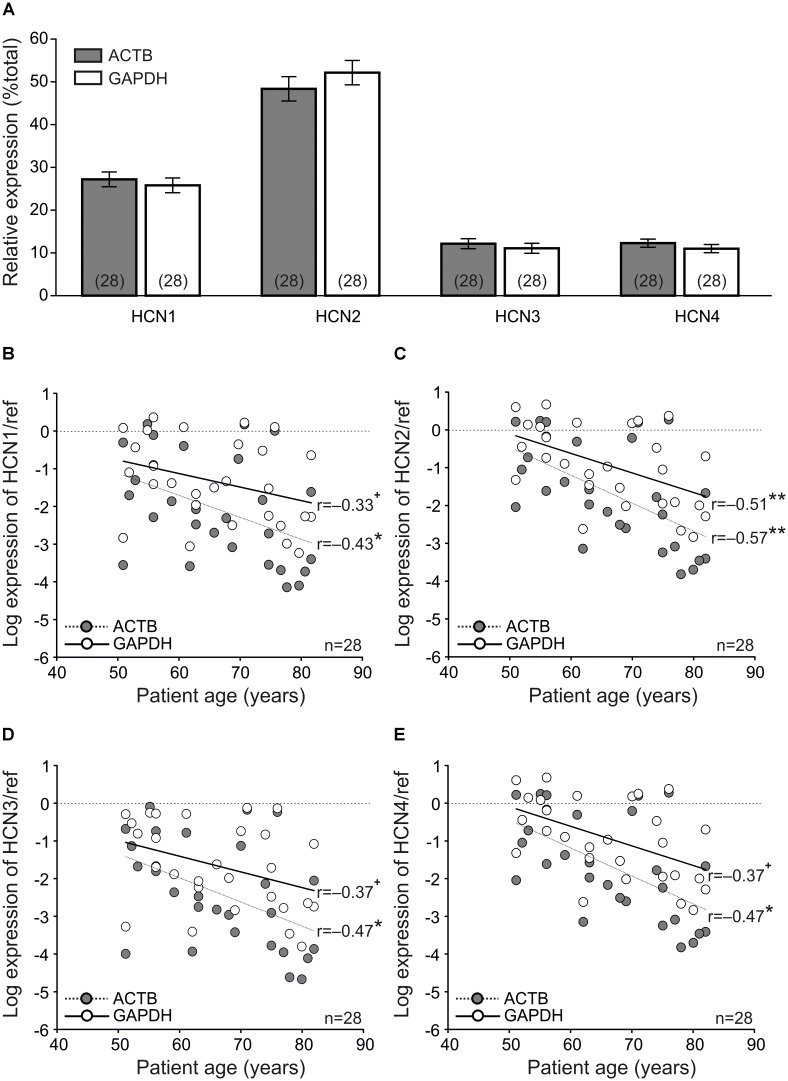
Quantitative PCR indicates age-dependent down-regulation of HCN channels. **(A)** Relative expression of HCN channel isoforms indicating that HCN2 is the predominant isoform on the transcriptional level. **(B–E)** Scatter plots showing the mRNA expression of HCN1-4 relative to the reference genes ACTB (gray symbols) and GAPDH (open symbols). Note the high concordance of relative expression values using both reference genes ( ^+^*P* < 0.1, paired *t*-test; ^∗^*P* < 0.05, paired *t*-test; ^∗∗^*P* < 0.01, paired *t*-test).

We have demonstrated so far that HCN channel inhibition leads to tonic contractions and enhances phasic activity in human detrusor smooth muscle. Next, we aimed to explore the HCN channel isoforms expressed in the human urinary bladder. To this end, we performed quantitative real-time reverse-transcriptase polymerase chain reaction (qPCR) analyses for all HCN1-4 isoforms using β-actin (ACTB) and glyceraldehyde-3-phosphate dehydrogenase (GAPDH) as reference genes (Kirschstein et al., 2015). As shown in **Figure 3A**, the results based on both reference genes were highly concordant indicating that HCN2-transcripts accounted for ∼50% of the HCN messenger RNA, followed by HCN1-transcripts (∼25%) and HCN3 as well as HCN4 (both ∼12%).

Although there were small differences between the two housekeeping genes, the correlation analyses showed that all isoforms were negatively related to patient age (**Figures 3B–E**). When using β-actin as the reference, the relationship between mRNA levels of all HCN isoforms showed a significantly negative correlation with age. However, a significant correlation with both reference genes was only seen for the predominantly expressed isoform HCN2 (*r* = -0.51 and *r* = -0.57, *P* < 0.01, paired *t*-test, *n* = 28 patients; **Figure 3C**).

**FIGURE 4:**
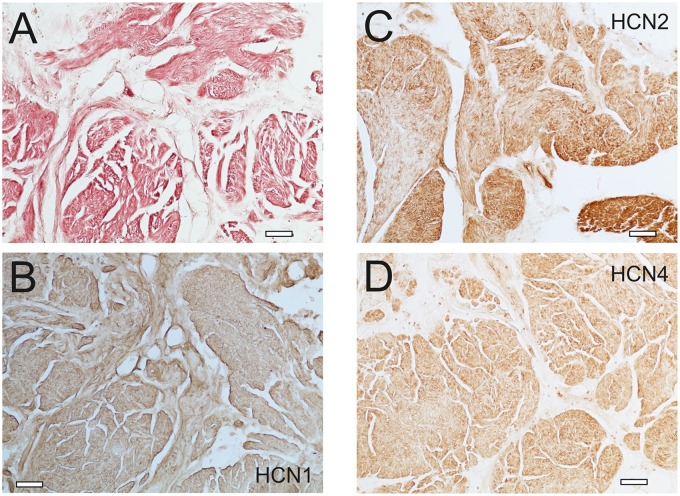
Immunohistochemistry indicates HCN channel expression on smooth muscle tissue. **(A)** Van Gieson staining of a human bladder specimen (patient #2 in **Table 2**) demonstrates the presence of smooth muscle tissue. **(B–D)** The immunohistochemical labeling of HCN1, 2 and 4 suggests that all these isoforms are present on smooth muscle tissue. Scale bar 100 μm.

To further explore the expression of HCN channels in the human urinary bladder, we performed immunohistochemistry with antibodies against HCN1, HCN2 and HCN4 (**Figure 4**) using samples of seven patients (**Table 2**). HCN1-immunoreactivity was observed in specimens from all patients (**Figure 4B**), and could be attributed to both smooth muscle tissue and connective tissue, in particular nerve fibers and blood vessels. However, the HCN2 expression was heterogeneous among different patients (**Table 2** and **Figure 4C**), and HCN2-immunoreactivity was predominantly observed in smooth muscle tissue and less so in connective tissue. Moreover, nerve fibers were almost HCN2-negative. Interestingly, the HCN4 expression entirely paralleled the HCN2-immunoreactivity (**Table 2** and **Figure 4D**), resulting in a strong and significant correlation (*r* = 0.94, *P* < 0.01, paired *t*-test; **Table 3**). It is important to note that there was no correlation between immunoreactivity and patients’ age (HCN1: *r* = 0.13; HCN2: *r* = 0.30; HCN4: *r* = 0.44; *P* > 0.3 for all, paired *t*-test, *n* = 7 patients), but the expression of all three isoforms appeared to be correlated to each other (**Table 3**).

**Table 3:** Spearman rank order correlation of immunohistochemical HCN isoform expression.

	HCN2	HCN4
HCN1	*r* = 0.626 (*P* = 0.096, *n* = 7)	*r* = 0.600 (*P* = 0.121, *n* = 7)
HCN2	–	*r* = 0.939 (*P* < 0.001, *n* = 7)


**FIGURE 5:**
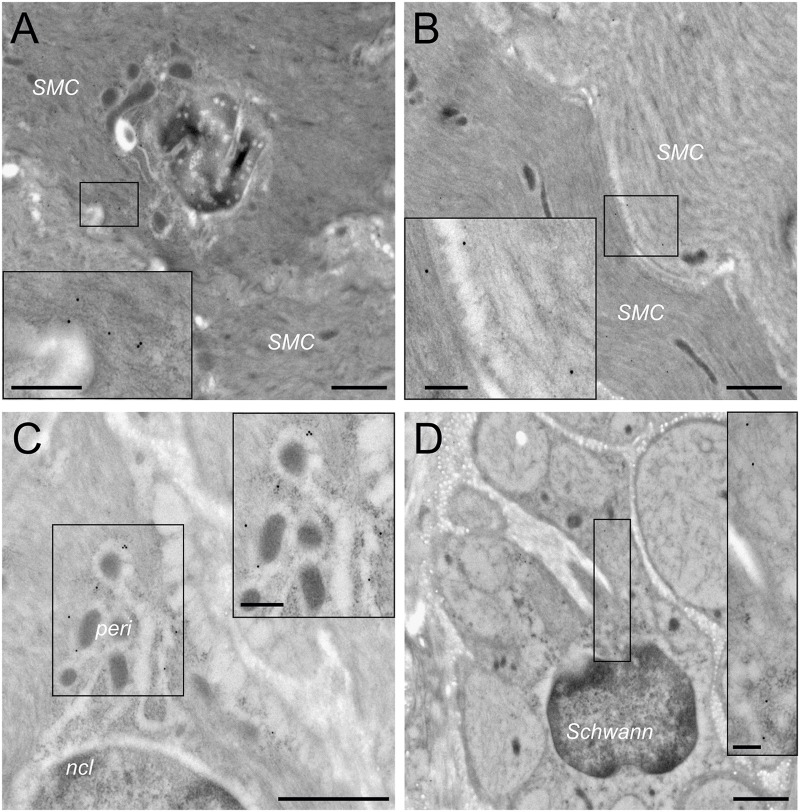
Transmission electron microscopy confirms HCN2 presence on smooth muscle cells. **(A)** Typical example of HCN2 immunogold particles asymmetrically located at the border between two smooth muscle cells (SMC; magnification 7.000×, scale bar 1 μm). In the inset taken from the small box (magnification 20.000×, scale bar 250 nm), immunogold particles are indicated by white arrows. **(B)** Occasionally, HCN2 immunogold particles were detected symmetrically at the border between two smooth muscle cells (magnification 7.000×, scale bar 1 μm). The inset (magnification 20.000×, scale bar 250 nm) shows immunogold particles indicated by white arrows. **(C)** In addition to the plasma membrane compartment, HCN2 immunogold particles were also detected in the perinuclear compartment (peri) around the nucleus (ncl) containing mitochondria and rough endoplasmic reticulum (magnification 12.000, scale bar 1 μm). The inset (magnification 20.000×, scale bar 250 nm) shows immunogold particles on the endoplasmic reticulum indicated by white arrows. **(D)** In peripheral nerves, HCN2 immunogold particles were only detected on Schwann cells, but largely absent from the unmyelinated axons (magnification 7.000×, scale bar 1 μm). The inset (magnification 20.000×, scale bar 250 nm) shows immunogold particles within a Schwann cell cytoplasm indicated by white arrows.

In order to confirm the presence of HCN channels on smooth muscle cells, we performed TEM with immunogold labeling for HCN2 in rat urinary bladder. As shown in **Figure 5A**, immunogold particles were found near the plasma membrane of smooth muscle cells, but the HCN2 expression differed substantially between individual smooth muscle cells (**Figure 5B**). Using higher magnification, we confirmed the presence of immunogold particles on the rough endoplasmic reticulum within the perinuclear compartment of smooth muscle cells (**Figure 5C**). With respect to nerve fibers, HCN2-immunogold particles were present only on Schwann cells, but not in the axonal compartment (**Figure 5D**).

### HCN1 Channels Contribute to the HCN-Dependent Motility in Murine Detrusor

**FIGURE 6:**
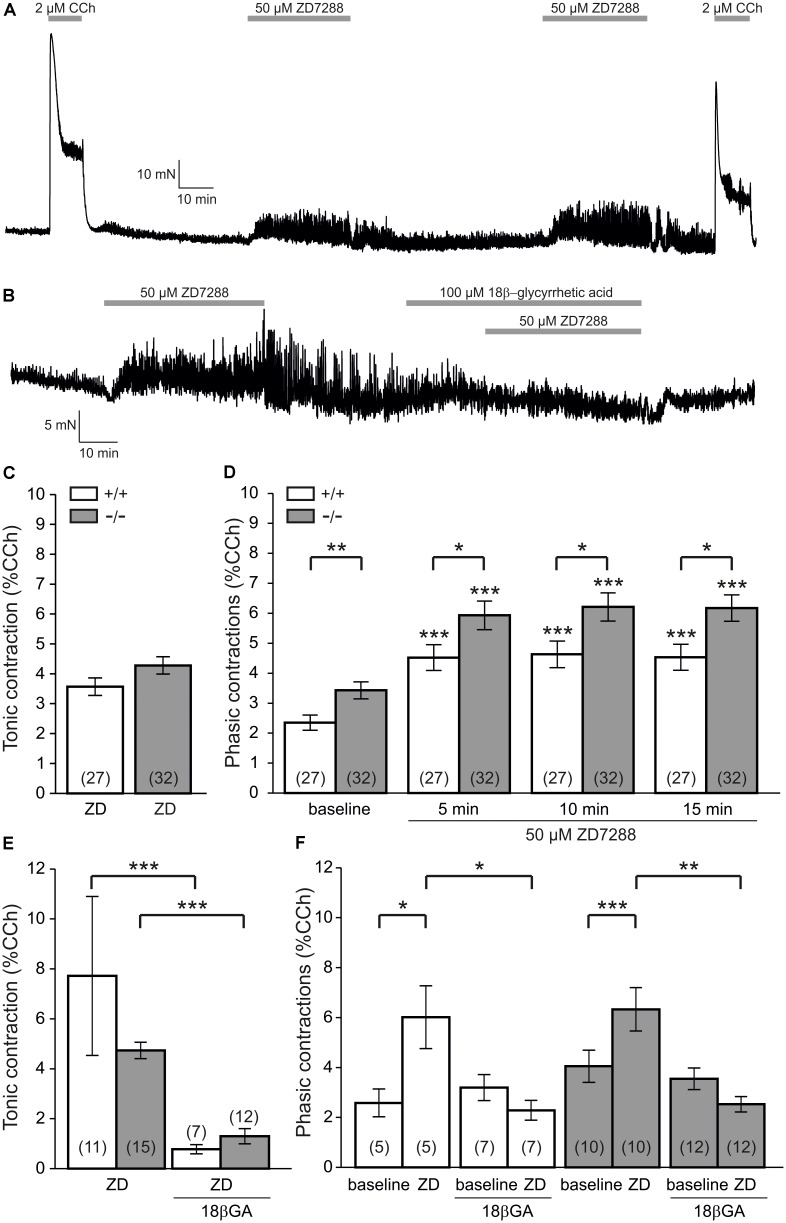
HCN1 contributes to the ZD7288-induced enhancement of phasic contractions. **(A)** Sample force recording showing the carbachol (CCh) and ZD7288-evoked response in a mouse bladder specimen. **(B)** Sample trace showing that pre-treatment with 18β-GA largely inhibited the ZD7288-induced tonic contraction as well as the enhancement of phasic contractions. **(C)** Tonic contractions by ZD7288 were not different between HCN1-deficient mice and wildtype littermates. **(D)** ZD7288 significantly enhanced phasic contraction amplitudes in both HCN1-knockout mice and controls (*P* < 0.001, Wilcoxon signed rank test). However, this increase was significantly more pronounced in HCN1-deficient mice (*P* < 0.05, Mann–Whitney test). Note that phasic contraction amplitudes were significantly higher in HCN1-knockout mice under baseline conditions (i.e., before ZD7288 application, *P* < 0.01, Mann–Whitney *U*-test). **(E)** Tonic contractions were significantly reduced by 18β-GA (*P* < 0.001, Mann–Whitney *U*-test), but without differences between HCN1^+/+^ and HCN1^-/-^ genotypes. **(F)** The ZD7288-induced enhancement of phasic contractions was significantly sensitive to 18β-GA, again similar in both genotypes.

Since no subtype-specific blockers for HCN channels are available, we next aimed to confirm the ZD7288-mediated effects in murine detrusor smooth muscle specimens in order to study HCN1-deficient mice. Detrusor specimens from wildtype (WT) mice showed exactly the same response to ZD7288 as did human preparations (**Figure 6A**). HCN channel inhibition caused robust tonic contractions that did not differ between both genotypes which suggests that there is no specific role of HCN1 in tonic contractions (WT: 3.6 ± 0.3%, *n* = 27; HCN1^-/-^: 4.3 ± 0.3%, *n* = 32; **Figure 6C**). However, the increase of phasic contraction amplitudes were significantly stronger in HCN1^-/-^ mice as compared to littermate controls indicating that HCN1 did contribute to the ZD7288-mediated enhancement of phasic contractions (WT after 15 min: 4.5 ± 0.4%, *n* = 27; HCN1^-/-^ after 15 min: 6.2 ± 0.4%, *n* = 32; *P* < 0.05; Mann–Whitney *U*-test; **Figure 6D**).

We then aimed to test whether gap junctions are again functionally involved in ZD7288-induced effects in the murine detrusor. To this end, we extended our experiments and applied the gap junction blocker 18β-GA (100 μM). In this subset of experiments, we found that 18β-GA almost abolished both the tonic contraction induced by ZD7288 (**Figures 6B,E**) and the enhancement of phasic contractions (**Figures 6B,F**). Moreover, there was no residual difference between HCN1^-/-^ (gray bars in **Figures 6E,F**) and control littermates (open bars in **Figures 6E,F**) indicating that residual contractility after ZD7288 was HCN1 channel independent.

### Lamotrigine Relaxes Detrusor Smooth Muscle

**FIGURE 7:**
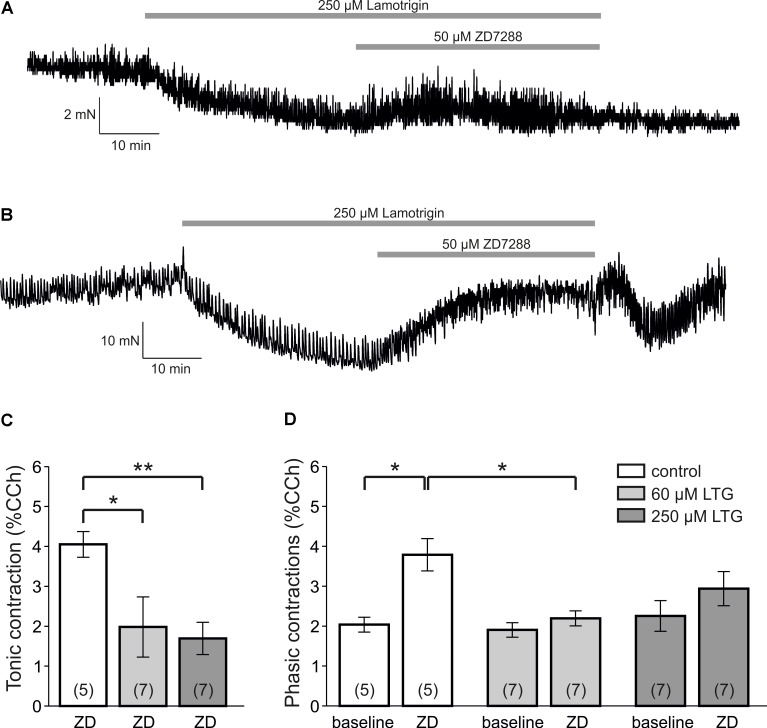
Lamotrigine-induced relaxation of the bladder is reversed by ZD7288. **(A)** Sample trace showing a force recording of a mouse bladder specimen that is challenged with 250 μM lamotrigine (LTG) leading to a reduced tone. In the presence of LTG, 50 μM ZD7288 partly reverses this tonic relaxation. **(B)** Sample trace of a human bladder specimen also showing that LTG-induced relaxation is reversed by ZD7288. **(C)** The tonic contraction in mouse bladder induced by ZD7288 is significantly reduced by pre-treatment with LTG (60 μM and 250 μM, ANOVA with Holm–Sidak *post hoc* test). **(D)** The ZD7288-induced enhancement of phasic contraction amplitudes in mouse bladder is abolished by pre-treatment with LTG (*P* < 0.05, Mann–Whitney *U*-test).

Lamotrigine (LTG) has been demonstrated to activate HCN channels (Poolos et al., 2002; Postea and Biel, 2011). We therefore asked whether LTG would relax detrusor smooth muscle cells and whether ZD7288 could reverse this relaxation (**Figures 7A,B**). In mouse bladder specimens, LTG caused a dose-dependent relaxation (60 μM: -2.2 ± 0.4 mN, *n* = 7 mice; 250 μM: -3.3 ± 0.8 mN, *n* = 7 mice; *P* < 0.05, ANOVA with Holm–Sidak *post hoc* test; **Figure 7A**). In human bladder strips, we could confirm the tonic relaxation induced by 250 μM LTG (12.0 ± 2.8 mN, *n* = 2 patients with 8 specimens; **Figure 7B**). This LTG-induced relaxation, in turn, was partly reversed by ZD7288, but ZD7288-induced contraction was significantly lower in the presence of LTG than under control conditions (**Figure 7C**; *P* < 0.05, ANOVA with Holm–Sidak *post hoc* test). Thus, LTG reduced the ZD7288-indced tonic contraction. Moreover, LTG abolished the ZD7288-induced increase of phasic contraction amplitudes (*P* < 0.05, Mann–Whitney *U*-test, **Figure 7D**).

## Discussion

The present study was conducted in order to explore the role of hyperpolarization-activated cAMP-gated non-selective (HCN) channels in detrusor smooth muscle contractile activity. By means of pharmacological inhibition as well as genetic ablation of the isoform HCN1 we could demonstrate a functional role of HCN channels in regulating tonic and phasic contraction amplitudes. Immunohistochemical analyses showed expression of all isoforms (HCN1-4) in human detrusor tissue. In addition, ultrastructural immunogold labeling confirmed the localization of HCN2 on detrusor smooth muscle cells. Since the commonly used anticonvulsant drug lamotrigine is acting as an agonist at HCN channels, lamotrigine-induced detrusor relaxation might be of clinical relevance.

### Frequency of Spontaneous Phasic Detrusor Contractions

Probably the most widely known function of HCN channels is the diastolic depolarization in the sinoatrial node (Scicchitano et al., 2012) leading to the bradycardia-inducing effects of the HCN channel blocker ZD7288 (Briggs et al., 1994). In the present study, we could not observe changes in the frequency of spontaneous phasic detrusor contractions in the mouse, different from human tissue. There are only few reports that have addressed the effects of ZD7288 on detrusor smooth muscle (Green et al., 1996; He et al., 2012; Deng et al., 2015; Kashyap et al., 2015). ZD7288 was found to increase the frequency in the rat detrusor (He et al., 2012; Deng et al., 2015). Importantly, both studies also used mucosa-free tissue and molecular data have indicated a substantial expression of HCN channels in mucosal tissue (He et al., 2012). Hence, the intact urothelium and lamina propria in murine tissue may have masked potential frequency changes by ZD7288. In contrast, in our human specimens, we only used detrusor smooth muscle, i.e., without adjacent mucosal and lamina propria tissue and were able to obtain frequency changes. Urothelial cells in particular have specialized signal pathways involved in detrusor contractility (Birder, 2006). Mucosal cells are capable to release various signaling molecules in response to physical stimuli such as stretch (Kumar et al., 2004). One of these is adenosine triphosphate (ATP) which is accepted to be instrumental in micturition activating ionotropic P2X3 receptors on suburothelial afferent nerve fibers (Burnstock, 2007). ATP is degraded to ADP which is an agonist of urothelial P2Y6 receptors and thus also involved in bladder contractility (Timóteo et al., 2014). In addition, ATP may influence myofibroblasts in the lamina propria leading to spontaneous depolarizations (Wu et al., 2004; Sui et al., 2006). Since HCN channels have been found on ICC in mouse colon (Shahi et al., 2014), mouse antrum (Si et al., 2012), rat detrusor (He et al., 2012) and human detrusor (Xue et al., 2012), it is very likely that HCN channels contribute to the pacemaker properties of these cells albeit inversely to their pacemaker role in the sinoatrial node.

### ZD7288-Mediated Increase of Tonic and Phasic Detrusor Contractions

One major finding of our study is the ZD7288-mediated enhancement of tonic and phasic detrusor contractions in both murine and human detrusor specimens. Occasionally, human preparations presented during baseline with strong spontaneous contractility with high amplitudes of phasic contractions, and these specimens showed a decrease rather than an increase of phasic contraction amplitudes in the presence of ZD7288. These observations may indicate that detrusor motility could be regulated by HCN channels in two opposing directions. Such a bidirectional modulation of activity has been demonstrated for ZD7288 in the globus pallidus (Chen et al., 2015). In the majority of the few organ bath studies available so far using rat detrusor smooth muscle and ZD7288, tonic contractions were not assessed or not discussed (Green et al., 1996; He et al., 2012; Deng et al., 2015). However, sample traces in the latter report studying the rat overactive bladder showed an enhanced tone in the presence of ZD7288 (Deng et al., 2015). In an earlier study, phasic contractions were enhanced by ZD7288 in an dose-dependent manner (Green et al., 1996), and more recently, Kashyap and co-workers also observed a concentration-dependent increase of tonic and phasic contraction amplitudes by ZD7288 using rat detrusor strips with intact urothelium (Kashyap et al., 2015). Hence, it seems to be a consistent finding that HCN channel inhibition activates rat detrusor contractility leading to enhanced tonic (Deng et al., 2015) and phasic contraction amplitudes (Green et al., 1996; Kashyap et al., 2015), as well as in murine and human tissue (present study).

In our hands, the ZD7288-mediated enhancement of tonic and phasic contractions was quite slow and developed over several minutes. On the cellular level, ZD7288 effects were described as slowly developing (BoSmith et al., 1993; Ghamari-Langroudi and Bourque, 2000; Stieber et al., 2005). Importantly, the delay in the organ bath is consistent with the lipophilic structure of this compound which inhibits the channel acting from the intracellular side and thus first has to pass through the cell membrane when administered extracellularly (Gasparini and DiFrancesco, 1997).

How could the contraction-enhancing effects of HCN channel blockade be explained? One potential mechanism relates to Ca^2+^-activated K^+^ (K_Ca_) channels. Ca^2+^ influx through voltage-gated Ca^2+^ channels activates both K_Ca_1 (BK) and K_Ca_2 (SK) channels (Herrera and Nelson, 2002), which play an important role in regulating the amplitudes of phasic and probably also tonic contractions (Trivedi et al., 1995; Herrera et al., 2000; Koh et al., 2012). Since HCN channels are colocalized with K_Ca_ channels on ICC (Lees-Green et al., 2011) and ZD7288 inhibits Ca^2+^ influx (Yu et al., 2004), it is conceivable that both channels interact in these cells and thus regulate detrusor motility. Although there is evidence that ZD7288 might directly block T-type Ca^2+^ channels at higher concentrations (Felix et al., 2003), at least two observations in the present study argue against the possibility that such unspecific effects have substantially contributed to the ZD7288-mediated increase of contraction amplitudes. On the one hand, the HCN channel agonist lamotrigine (Poolos et al., 2002; Postea and Biel, 2011) might also block T-type Ca^2+^ channels (Hainsworth et al., 2003), but led to detrusor relaxation, i.e., the opposite effect of ZD7288, confirming a previous study (Kashyap et al., 2015). On the other hand, our findings on HCN1-deficient mice demonstrate that at least the effects of HCN1 on phasic contraction amplitudes cannot be explained by such an unspecific effect of ZD7288. Interestingly, K_Ca_1 channels have been found to be down-regulated in HCN1 knock-out mice (Bishao et al., 2015), thereby again emphasizing the role of K_Ca_ channels for phasic contractions. In addition, there are at least two further groups of ion channels that may be involved in phasic contractility. First, inhibition of voltage-gated K_v_7 (KCNQ) channels has been demonstrated to increase smooth muscle phasic contraction amplitudes of smooth muscle cells (Anderson et al., 2013). Second, the Ca^2+^-activated Cl^-^ channel anoctamin-1 is also believed to play a role in pacemaker cells such as ICC (Zhu et al., 2009) and phasic contractions were reduced by anoctamin-1 blockade (Bijos et al., 2014).

### Cellular Localization of HCN Channels in the Urinary Bladder

Recent work has presented evidence for the presence of HCN channels on ICC in the rat and human urinary bladder (He et al., 2012; Xue et al., 2012), and ICC are connected with smooth muscle cells via gap junctions (Huizinga et al., 1997; Daniel and Wang, 1999; Nemeth et al., 2000; Ward et al., 2000). Therefore, we have used the gap junction blocker 18β-glycyrrhetic acid (18β-GA) in order to isolate the ZD7288-mediated effects without contamination by ICC-smooth muscle connections. Under these conditions, the ZD7288 no longer caused a significant increase of phasic contraction amplitudes, but spontaneous contractions were not abolished indicating the predominant role of HCN channels on ICC in modulating phasic contraction. This finding is consistent with observations that 18β-GA application reduced pacemaker potentials (Santicioli and Maggi, 2000) as well as spontaneous contractions in a cystitis mouse model (Okinami et al., 2014). However, 18β-GA did not significantly change the frequency of phasic contractions nor did it occlude the ZD7288-mediated increase of phasic contraction frequency. This suggests that pacemaker cells other than ICC such as platelet derived growth factor receptor-α (PDGFRα) expressing pacemaker cells (Lee et al., 2013, 2014) and/or intercellular connections other than gap junctions are involved in these processes.

With respect to the isoform, our data on HCN1 knock-out mice revealed that phasic contractions presented with higher contraction amplitudes compared to wildtype mice, but the increased tonic contraction in the presence of ZD7288 was not altered in HCN1-deficient mice. This supports the view that HCN1 may be a major HCN channel subtype on ICC involved in phasic contraction frequency regulation and is consistent with previous reports that have identified HCN1 on ICC (He et al., 2012; Liu et al., 2017).

In contrast to phasic contractions, the tonic contraction in the presence of ZD7288 was significantly reduced, but not prevented suggesting that HCN channels may be directly expressed on smooth muscle cells and limit detrusor baseline tone. This view is supported by the functional presence of an inwardly rectifying ZD7288-sensitive ion channel on rat bladder smooth muscle cells (Green et al., 1996). Here, we could demonstrate by immunogold staining that HCN2 is expressed on the plasma membrane of smooth muscle cells, but not on axons. In line with this finding, the Na^+^ channel blocker tetrodotoxin (TTX) had no effect on the ZD7288-mediated contraction suggesting that intrinsically active nerve fibers expressing voltage-gated Na^+^ channels (Black et al., 2003) do not play a major role in HCN channel modulation of detrusor motility. This observation confirms previous studies in guinea-pig and rat (Zagorodnyuk et al., 2009; Hammad et al., 2014; Kashyap et al., 2015) and suggests that efferent nerve fibers innervating ICC and/or smooth muscle cells do not express a relevant number of HCN channels. In contrast, voltage-gated Na^+^ channels have been found on both smooth muscle cells (Nakajima et al., 2008; Meguro et al., 2009; Zhu et al., 2010; Ho et al., 2013), and ICC (Cheng et al., 2012). A limitation certainly is that these results do not rule out a role of TTX-insensitive Na^+^ channels which may even be expressed on smooth muscle cells (Yoshida, 1994). In particular, spontaneous contractions were found to be insensitive to TTX, while contractions evoked by electrical stimulation were blocked by TTX (Sugaya and de Groat, 2000; Longhurst and Uvelius, 2001; De Bock et al., 2011). A further limitation is that silent nerve fibers requiring external stimulation could not be tested by this procedure.

By quantitative RT-PCR, we identified HCN2 as the predominant isoform in human tissue, in contrast to previous work pointing to HCN4 (Xue et al., 2012; Kashyap et al., 2015). Certainly, human tissue may always be altered by the underlying disease that may have an impact on the expression of housekeeping genes (Kirschstein et al., 2015), but all studies used tumor-free samples from cystectomy patients suffering from bladder cancer. Thus, further studies with new patient cohorts are needed to clarify these uncertainties.

### Clinical Relevance of HCN Channel Modulation of Detrusor Contractility

With respect to the clinical relevance of our study, we firstly found that the HCN channel modulation of detrusor contractility was age-dependent. On the transcriptional level, there was a down-regulation in aged bladder specimens, which, in contrast, was not paralleled on the protein level. More importantly, however, the effects of ZD7288 on phasic contractions were significantly more pronounced in specimens from the elderly. This age-dependent effect of HCN channel inhibition is a novel finding, but fits perfectly to other age-dependent changes of detrusor contractility (Wuest et al., 2005). Hence, these age-dependent effects on the functional level appear to be most clinical relevant and further studies should address the specific role of these channels in overactive bladder.

Secondly, we obtained evidence that lamotrigine led to dose-dependent detrusor relaxation. Lamotrigine (LTG) has been demonstrated to activate HCN channels (Poolos et al., 2002; Postea and Biel, 2011), and ZD7288 could reverse the lamotrigine-induced tonic relaxation, therefore, we attribute the lamotrigine effect to a direct activation of HCN channels on smooth muscle cells. Since LTG is a commonly used anticonvulsant drug, and 60 μM is achieved in the patient serum (Johannessen et al., 2003; Beck et al., 2006), this finding could be particularly relevant in patients suffering from epilepsy and concomitantly from overactive bladder. Overactive bladder is a very common condition in elderly patients and the current treatment options are unsatisfactory in many cases (Hampel et al., 2017). In fact, LTG was shown to be beneficial in overactive bladder, whereas the effects have been attributed to an inhibition of Na ^+^ channels on nerve fibers (Loutochin et al., 2012). Gabapentin, another anticonvulsant drug commonly used for focal epilepsies has been demonstrated to activate HCN channels (Surges et al., 2003) again opening the therapeutic opportunity to address overactive bladder in epileptic patients simultaneously.

## Author Contributions

TK, RK, and OH contributed conception and design of the study. FM, SM, LK, AS, KP, SR, TW, MF, and TK performed experiments. TK, KK, and CP organized the database. FM, SM, LK, KP, and TK performed the statistical analysis. TK wrote the first draft of the manuscript. CP, MF, OH, and RK wrote sections of the manuscript. AS, KK, KP, and SR contributed to manuscript preparation. All authors contributed to manuscript revision, read and approved the final version of this manuscript for submission.

## Conflict of Interest Statement

The authors declare that the research was conducted in the absence of any commercial or financial relationships that could be construed as a potential conflict of interest.
